# On Aneurism and Its Cure by a New Operation

**Published:** 1829-07-01

**Authors:** 


ON ANEURISM, AND ITS CURE BY A NEW OPERATION. Dedicated by Permission to the King. By *James Wardrop*, Surgeon to His Majesty. Octavo, pp. 117. Longman and Co. 1829.

IN contrasting the modern surgery of aneurism with the treatment of it a century ago, it is gratifying to observe how much science has done for the alleviation of a disease, which at an earlier period must have proved almost always fatal. Amputation no doubt afforded occasional relief, but in aneurisms of the great arteries, such as the axillary and external iliac, surgery afforded little or no resources; and if Nature did not, in a few rare instances, manifest her extraordinary powers by a spontaneous cure, death alone terminated a miserable train of sufferings.

To English surgeons almost exclusively belongs the merit of having raised the pathology of the arterial system to its present improved condition, for notwithstanding the attempts made, particularly on the Continent, to deprive him of the honour, it was Hunter who first established practically those principles which afterwards emboldened an Abernethy and a Cooper to apply ligatures upon the great arterial trunks, the integrity of which was at one time deemed indispensable to the existence of the organs they supply.

In the present volume, after the preliminary observations in which the nature, formation, terminations, &c. of aneurism are detailed, Mr. Wardrop proceeds to give an historical view of its pathology and treatment by Hunter and his successors. It was generally supposed by them, and even some modern surgeons, that, in cases where a ligature is applied to an artery on the cardiac side of an aneurism, the circulation is completely suspended in the sac. Sir Everard Home, however, observed that this was not the fact; but, on the contrary, that the circulation, though interrupted, was still partially continued, and that this diminution in the force of the circulation, was alone sufficient to ensure, sooner or later, the coagulation and subsequently the consolidation of the blood in the sac. This fact, Mr. Wardrop states, "enables us to establish a new principle for operating in aneurisms so situated, as hitherto to have been considered beyond the reach of art, and to which the Hunterian principle of operating is totally inapplicable."

The operation here alluded to by Mr. Wardrop consists in applying the ligature to the artery on the *distal* instead of the *cardiac* side of the aneurismal tumour. The operation was suggested by Brasdor, Professor of Surgery in the Paris School, forty years ago, and was subsequently executed, but without success, by Deschamps, in a case of aneurism of the femoral artery close to Poupart's ligament. Finally, the operation was performed in this country by Sir A. Cooper, in a case of aneurism of the external iliac artery, with the same unfavourable result. It was then abandoned, and some of the arguments adduced against its repetition were, that a ligature on the distal side of an aneurismal tumour would cause the blood to accumulate in the sac, and thereby risk its being mechanically burst by the force of the circulation; or that the attenuation of its walls by distention would excite inflammation of the sac which occasionally proves fatal.

Now Mr. Wardrop asserts, that the first objection is utterly groundless, and that the reverse is the fact.

"When the ligature (he observes) is placed on the distal side of the aneurism, we know from experience that there is immediately a diminution of the bulk of the tumour. The fluid blood can find in such a case a ready exit back from the sac into the trunk from whence it came, and thus again passes into the circulation, in place of, as in the other case, having to pass through capillaries into veins, and as Nature immediately finds a new channel, there is no more blood impelled into the tumour afterwards."

Mr. Wardrop gives four cases in illustration of the practicability of the operation. In the first he operated himself under the most unfavourable circumstances, the patient being 75 years of age, and applied the ligature on the distal side of an aneurism of the carotid artery with perfect success. An account of three similar cases follows, the two first of which were not so successful, but the last, which was performed by Dr. Bush, is said to have been completely successful. Having thus detailed the particulars of these operations, the author proceeds to make an application of his principles to the cure of aneurism of the arteria innominata. In doing so he observes, that the process employed by Nature in the spontaneous cure is precisely analogous to that which takes place in the cure by the Hunterian operation; for in both cases the coagulation of the blood proceeds from the circumference of the sac towards the centre. From this fact Mr. Wardrop was led to infer that "in cases of aneurism of the arteria innominata, the progress of the disease might be arrested by tying its two great branches, the carotid and subclavian; and although a certain portion of blood would still continue to pass along the innominata to those branches of the subclavian on the cardiac side of the ligature, the ligature being necessarily placed on the subclavian artery after it emerges from between the scaleni muscles, yet such would be the diminution of the impetus of the blood in the sac, that the process of thickening of its parietes would not only go on, and thus its future increase be prevented, but even a permanent obliteration of the aneurismal cavity would be accomplished." This view is supported by the fact that, in all cases of aneurism of any growth, we invariably find laminæ of coagula adhering to the internal surface of the sac. It is the process which Nature adopts to cure the disease, by gradually filling the sac with successive deposits, until at last the cavity be so completely plugged up, as no longer to admit a column of blood to pass through it. Now if we can diminish the column of blood passing through the sac of an aneurism, we most powerfully assist Nature in her efforts to consolidate the contents; for, according as the blood admitted through the sac is lessened in quantity, the greater will be the disposition in the blood contained in it to coagulate. To this some have objected that the collateral branches enlarge so much, that the usual quantity of fluid circulates through the sac. Here, again, past observation has been deficient; for, as Mr. Wardrop observes, though the collateral vessels do enlarge most considerably, immediately after the application of the ligature, yet in a very short time they again diminish in volume, though not quite to their original diameters.

These views founded on the pathology of the disease, and the observation of the phenomena of spontaneous cure, emboldened the author to put his plan into execution in a well-marked case of aneurism of the arteria innominata. In this case the pulsation of the right carotid, with its several branches, was not perceptible, so that its obliteration was presumed. A ligature was therefore applied to the right subclavian, and though many untoward derangements of general health retarded its progress, the cure is said to be complete; for, at the last report, the only appreciable remains of the aneurismal tumour, which originally was perceptible at the root of the neck, was an unnatural feeling of hardness above the clavicle. In this case, Nature had considerably advanced in the spontaneous cure by the obliteration of the canal of the carotid, so that art was only required to assist by obliterating that of the subclavian. In those instances, however, of aneurism of the arteria innominata, where both the carotid and subclavian are quite pervious, Mr. Wardrop advises the application of the ligature to the carotid artery first, and to the subclavian at a subsequent period. His view on this subject seems to have been attended with a more favourable result than could possibly have been anticipated; for, in a case of aneurism of the arteria innominata, lately operated on by Mr. Evans, of Belper in Derbyshire, the ligature of the carotid was alone sufficient for the cure, the subclavian having become *spontaneously* obliterated eight or ten days after the operation.

